# Symptom remission at 12-weeks strongly predicts long-term recovery from the first episode of psychosis

**DOI:** 10.1017/S0033291719001399

**Published:** 2020-07

**Authors:** Paola Dazzan, Julia M. Lappin, Margaret Heslin, Kim Donoghue, Ben Lomas, Uli Reininghaus, Adanna Onyejiaka, Tim Croudace, Peter B. Jones, Robin M. Murray, Paul Fearon, Gillian A. Doody, Craig Morgan

**Affiliations:** 1 Department of Psychosis Studies, Institute of Psychiatry, Psychology & Neuroscience, King's College London, London, UK; 2 National Institute for Health Research Mental Health Biomedical Research Centre at South London and Maudsley NHS Foundation Trust and King's College London, London, UK; 3 School of Psychiatry, Faculty of Medicine, University of New South Wales, Sydney, Australia; 4 Department of Health Service & Population Research, Institute of Psychiatry, Psychology & Neuroscience, King's College London, London, UK; 5 Department of Addictions, Institute of Psychiatry, Psychology & Neuroscience, King's College London, London, UK; 6 Department of Psychiatry, University of Nottingham, Nottingham, UK; 7 Department of Public Mental Health, Central Institute of Mental Health, Medical Faculty Mannheim, Heidelberg University; 8 School of Nursing & Health Sciences, University of Dundee, Dundee, UK; 9 University of Cambridge, and Cambridgeshire and Peterborough NHS Foundation Trust, Cambridge, UK; 10 Discipline of Psychiatry, School of Medicine, Trinity College, Dublin, Ireland

**Keywords:** Clinical outcome, functional outcome, psychosis, remission, schizophrenia

## Abstract

**Background:**

To determine the baseline individual characteristics that predicted symptom recovery and functional recovery at 10-years following the first episode of psychosis.

**Methods:**

AESOP-10 is a 10-year follow up of an epidemiological, naturalistic population-based cohort of individuals recruited at the time of their first episode of psychosis in two areas in the UK (South East London and Nottingham). Detailed information on demographic, clinical, and social factors was examined to identify which factors predicted symptom and functional remission and recovery over 10-year follow-up. The study included 557 individuals with a first episode psychosis. The main study outcomes were symptom recovery and functional recovery at 10-year follow-up.

**Results:**

At 10 years, 46.2% (*n* = 140 of 303) of patients achieved symptom recovery and 40.9% (*n* = 117) achieved functional recovery. The strongest predictor of symptom recovery at 10 years was symptom remission at 12 weeks (adj OR 4.47; CI 2.60–7.67); followed by a diagnosis of depression with psychotic symptoms (adj OR 2.68; CI 1.02–7.05). Symptom remission at 12 weeks was also a strong predictor of functional recovery at 10 years (adj OR 2.75; CI 1.23–6.11), together with being from Nottingham study centre (adj OR 3.23; CI 1.25–8.30) and having a diagnosis of mania (adj OR 8.17; CI 1.61–41.42).

**Conclusions:**

Symptom remission at 12 weeks is an important predictor of both symptom and functional recovery at 10 years, with implications for illness management. The concepts of clinical and functional recovery overlap but should be considered separately.

## Introduction

Our ability to predict outcome following a first psychotic episode remains limited. Decades of research have shown that factors such as earlier age of onset, male gender, longer duration of untreated illness, and insidious onset are each grossly associated with worse outcome in schizophrenia (Johnstone *et al*., 1989; Jablensky *et al*., 1992; Leung and Chue, 2000; Marshall *et al*., 2005; Rabinowitz *et al*., 2006). None of these factors, however, has proven sensitive or specific enough to be clinically useful in predicting the outcome for an individual.

This lack of knowledge about clinically useful predictors reflects several issues. First, long-term cohort studies of first-episode psychosis (FEP) to date have evaluated prevalence samples with schizophrenia only (rather than all psychoses), which tend to have an over-representation of patients with poorer outcomes and an under-representation of those who do not remain in treatment (van Os *et al*., 1997). Second, many have implemented structured treatment protocols, not necessarily reflective of real-life practice, with consequent limited generalizability of findings. Third, there has been variability in the timeframes evaluated, the assessment tools used, and the outcome measures reported. Definitions used for remission and recovery, for example, range from the total absence of symptoms (Thara *et al*., 1994) to symptoms present below a certain threshold (Crumlish *et al*., 2009) for variable periods of time. Finally, symptom remission and social functioning have often been reported together, with both considered necessary for a good outcome, although such coupling lacks an empirical basis.

Evidence from short-term (up to 2 years) follow-up studies and clinical trials suggests that early improvement in symptom severity may predict response to antipsychotic drugs and short-term clinical outcome (Craig *et al*., 1999; Robinson *et al*., 1999; Emsley *et al*., 2006). Although symptomatic improvement following first treatment (4–6 weeks to 3 months) has been associated with better outcome at 1–2 years (Lieberman *et al*., 1993; Robinson *et al*., 1999), its relationship with longer-term outcome remains unclear. Follow-up studies of more than 8 years' duration have indirectly reported that non-specific measures of early remission, such as ‘prompt treatment’ (Friis *et al*., 2016) and ‘shorter proportion of time spent experiencing symptoms in first few years of illness’ (Wiersma *et al*., 1998; Harrison *et al*., 2001; Hegelstad *et al*., 2012) are associated with better long-term outcome. None of these studies has evaluated the role of time to the first remission according to operationalised criteria in long-term outcome.

In order to move towards a novel framework for targeted interventions, we need longitudinal data from unselected samples of incident cases of all psychoses (not just schizophrenia), evaluated using clear operational definitions of symptomatic and functional outcomes. Factors that predict sustained symptom remission and functional recovery should be explored separately to inform differential interventions. This is what we aimed to do in this unique study.

In a large cohort of individuals experiencing FEP, we evaluated predictors of symptom and functional remission and recovery over the subsequent 10-years in three domains (demographic, clinical, social) (Morgan *et al*., 2014).

## Method

We conducted a 10-year follow-up study (ÆSOP-10) of an epidemiological cohort of 557 individuals initially assessed at the time of their first episode of psychosis (FEP) in two UK centres, south-east London (urban) and Nottingham (semi-urban). This sample comprised all incident cases who presented to specialist mental health services within tightly-defined catchment areas in the two centres (*n* = 532). The study was approved by the local Research Ethics Committees.

Our procedures for tracing cases were in line with those of previous long-term follow-up studies of psychosis (Harrison *et al*., 2001; White *et al*., 2009). At approximately 10-years after inclusion, we sought to trace and re-interview all cases included in the baseline study (see Morgan *et al*., 2014).

### Baseline

At baseline, information was collated on clinical presentation and demographic and social characteristics. Assessments were completed on a range of clinical and social risk factors (Morgan *et al*., 2006). Baseline ICD-10 and DSM-IV diagnoses were determined at consensus meetings using data collected with the Schedules for Clinical Assessment in Neuropsychiatry (SCAN) (WHO, 1992). From this schedule, a series of symptom dimensions (mania, reality distortion, negative, depressive and disorganization) was obtained (Demjaha *et al*., 2009). We created an index of disadvantage by counting the presence of four markers: living alone, being single, being unemployed, living in rented housing.

### Symptom course and outcome

We used an extended version of the WHO Life Chart to collate information across symptom and social course and outcome, as previously described (Morgan *et al*., 2014). The Life Chart has been used successfully in previous long- term follow-up studies, including those with follow-up periods in excess of 10-years, and is designed to collate information from multiple sources (Sartorius *et al*., 1996; Morgan *et al*., 2014). We included additional substance use and service contact information, and a timeline to document psychotic symptoms and contacts with mental health services. We recorded prescription of, and compliance with, anti-psychotic medications throughout the follow-up. Although the Life Chart has been shown to produce reliable ratings (Susser *et al*., 2000), all clinical ratings were made by consensus.

Information on symptoms in the month preceding follow-up was collected using the Schedules for Clinical Assessment in Neuropsychiatry (SCAN) Version 2 (WHO, 1992), the Scale for the Assessment of Negative Symptoms (SANS), and the Global Assessment of Function (symptom score) (Endicott *et al*., 1976). Information from the Life Chart and SCAN were used to make a lifetime diagnosis.

### Symptom remission and recovery

In line with Andreasen and colleagues (Andreasen *et al*., 2005), we defined remission as absence of overt psychotic symptoms (operationalised as a score of 2 or 3 on Rating Scale 2 in the SCAN; 0 = absence, 1 = symptom occurred, but fleeting, 2 = symptom definitely present, 3 = symptom present more or less continuously) for a period of at least 6 months. We used the symptom criteria to define early symptomatic remission beginning at most 3 months after the first contact. We defined symptom recovery as sustained remission for 2 or more years.

### Social functioning and functional recovery

Information on sociodemographic markers of social function and integration across a number of domains (housing, employment, relationships, education, and social networks) over follow-up was collected using the Life Chart (Table 2). Information on social function in the month prior to follow-up was collected using the Life Chart and the GAF (disability score) (24). GAF score equal to or greater than 60 was used to define functional recovery.

### Analysis

We describe primary outcomes using frequencies and percentages, and means or medians and standard deviations or inter-quartile ranges. We examined associations between each set of putative baseline and early course predictors and (a) symptom recovery at 10 years using logistic regression and (b) functional recovery (i.e. GAF > 60) at 10 years using linear regression. We first fit univariable models and then adjusted (a) for study centre, age, gender, and ethnicity and (b) in turn, for other variables found to be associated with each outcome, retaining in the final models only those variables that improved model fit based on likelihood ratio tests. For final models, we further report pseudo-R^2^ statistics and percentage recovered correctly predicted, estimated using post-estimation commands in Stata. All analyses were conducted using Stata 13.

## Results

### Description of the sample

Of the original 532 incident cases, 37 (7.0%) had died, 29 (5.5%) had left the country and 8 (1.5%) were excluded based on information not available at baseline. Of the remaining 458 cases, we obtained useable information on symptom course and outcome across one or more of our three domains on 387 (84.5% of 458) for at least 8 years of follow-up. There were no systematic differences by age, gender, ethnicity, duration of untreated psychosis (DUP), baseline employment, baseline diagnosis, mode of initial contact with mental health services, or study centre between those with follow up information and those without (Morgan *et al*., 2014). The sample characteristics are presented in Table 1.

**Table 1.** Demographic, social, and clinical characteristics of the sample

	Total sample (*n* = 387)
	Median (IQR)
Age (years)	29.0 (22.0–36.0)
Duration of untreated psychosis (weeks)^a^	8.3 (2.0–32.2)
Length of follow-up (years)^b^	10.7 (9.8–11.4)
	No (%)
Study centre	
London	230 (59.4)
Nottingham	157 (36.1)
Gender	
Male	215 (55.6)
Female	172 (44.4)
Ethnicity	
White British	167 (43.2)
Black Caribbean	108 (27.9)
Black African	45 (11.6)
White Other	25 (6.5)
Asian	22 (5.7)
Other (all)	20 (5.2)
Place of birth^c^	
UK	288 (76.0)
Non-UK	91 (24.0)
Lifetime Substance Abuse/Dependence^d^	77 (22.9)
Lifetime Alcohol Abuse/Dependence^d^	54 (16.1)
Baseline diagnosis	
Schizophrenia	167 (43.2)
Mania	54 (14.0)
Depression	56 (14.5)
Schizoaffective	18 (4.7)
Brief	35 (9.0)
Other	57 (14.7)
Acute mode of onset^e^	157 (46.0)
Course Type^f^	
No further episodes	43 (12.5)
Episodic	69 (20.0)
Neither	153 (44.3)
Continuous	80 (23.2)
Recovered (symptoms)^g^	140 (46.2)
Recovered (functional)^h^	117 (40.9)
Employed at follow-up^i^	66 (22.8)
Marital status (follow-up)^j^	
Married/steady relationship	95 (31.7)
Single	205 (68.3)
Parental status (follow-up)^k^	
No children	164 (55.6)
Parent, lives with child	80 (27.1)
Parent, child lives with others	51 (17.3)

IQR, Interquartile range

a Missing, 14.

b Missing, 126.

c Missing, 8.

d Missing, 51.

e Missing, 46.

f Missing, 42.

g Missing, 84.

h Missing, 101.

i Missing, 98.

j Missing, 87.

k Missing, 92.

### Remission and symptom and functional recovery at 10 years

Details of illness course and symptom and social outcome have been previously reported (Morgan *et al*., 2014). Briefly, at follow-up 213 cases (65.3% of 326) were not experiencing psychotic symptoms and 140 (46.2% of 303 on whom complete data were available) met criteria for recovery. Relevant to this study, 77 (57.9%) of these had been in remission at 12-weeks, compared with 34 (22.8% of 163) of those who were not in recovery at 10-years (Table 2). Among cases for whom we had reliable medication information (228; 75.2% of 303 with data on recovery), 56.4% of those who were recovered (57 of 101) had been prescribed antipsychotic medication in the preceding 2-years, *v.* 85.8% (109 of 127) of those not recovered (χ^2^ 24.6, df 1, *p* < 0.001) (Note: all cases were, at some point, prescribed antipsychotic medication).

**Table 2.** Symptomatic Recovery at 10 years (unless specified, number included in analyses = 303)

	Not recovered (*N* = 163) *N* (%) or mean (s.d.)	Recovered (*N* = 140) *N* (%) or mean (s.d.)	Unadj. OR	95% CI	*p*	Adj. OR (1)^a^	95% CI	*p*
Demographic								
Study Centre								
London	109 (66.9)	94 (67.1)	1.00	–	–	1.00	–	–
Nottingham	54 (33.1)	46 (32.9)	0.99	0.61–1.60	0.960	0.63	0.36–1.14	0.128
Sex								
Men	95 (58.3)	67 (47.9)	1.00	–	–	1.00	–	–
Women	68 (41.7)	73 (52.1)	1.52	0.97–2.39	0.070	1.53	0.96–2.43	0.077
Age								
16–29	88 (54.0)	74 (52.9)	1.00	–	–	1.00	–	–
30–64	74 (46.0)	66 (47.1)	1.05	0.67–1.65	0.844	0.97	0.61–1.54	0.881
Ethnicity								
White British	53 (32.5)	66 (47.1)	1.00	–	–	1.00	–	–
Other	110 (67.5)	74 (52.9)	**0.54**	**0.34–0.86**	**0.010**	**0.43**	**0.25–0.76**	**0.003**
Social								
Index of social disadvantage^b^								
0, 1	21 (16.4)	37 (31.6)	1.00	–	–	1.00	–	–
2	31 (24.2)	30 (25.6)	0.55	0.26–1.14	0.110	0.53	0.24–1.18	0.121
3	40 (31.3)	30 (25.6)	**0.43**	**0.21–0.87**	**0.019**	**0.40**	**0.18–0.87**	**0.021**
4	36 (28.1)	20 (17.1)	**0.32**	**0.15–0.68**	**0.003**	**0.29**	**0.13–0.67**	**0.003**
Loss, separation from parent^c^								
None	56 (47.1)	58 (53.2)	1.00	–	–	1.00	–	–
Loss (parent died)	10 (8.4)	12 (11.0)	1.16	0.46–2.90	0.753	1.07	0.40–2.86	0.889
Separation from parent	53 (44.5)	39 (35.8)	0.71	0.41–1.24	0.226	0.70	0.39–1.28	0.245
Education^d^								
University	12 (7.6)	20 (14.5)	1.00	–	–	1.00	–	–
Further	50 (31.9)	31 (22.5)	0.37	0.16–0.87	0.022	0.48	0.20–1.19	0.114
GCSE	42 (26.8)	42 (30.4)	0.60	0.26–1.38	0.230	0.77	0.32–1.88	0.569
No qualifications	53 (33.8)	45 (32.6)	0.51	0.22–1.16	0.106	0.61	0.25–1.45	0.263
Early symptom course								
Remission at 12 weeks^e^								
No	115 (77.2)	56 (42.1)	1.00	–	–	1.00	–	–
Yes	34 (22.8)	77 (57.9)	**4.65**	**2.78–7.78**	**<0.001**	**4.47**	**2.60–7.67**	**<0.001**
% time on med, first 3 months^f^^,^^g^	65.9 (38.2)	66.8 (38.5)	1.00	0.99–1.01	0.844	1.00	0.99–1.01	
Clinical								
Mode of onset^h^								
Acute	55 (39.8)	67 (51.9)	1.00	–	–	1.00	–	–
Insidious	83 (60.1)	62 (48.1)	0.61	0.38–1.00	0.048	0.63	0.38–1.05	0.074
DUP^h^								
Short	70 (44.3)	82 (59.4)	1.00	–	–	1.00	–	–
Long	88 (55.7)	56 (40.6)	0.54	0.34–0.86	0.010	0.54	0.33–0.87	0.012
Diagnosis								
Non-affective	132 (81.0)	87 (62.1)	1.00	–	–	1.00	–	–
Mania	13 (8.0)	28 (20.0)	**3.27**	**1.60–6.66**	**0.001**	**3.22**	**1.54–6.73**	**0.002**
Depression	18 (11.0)	25 (17.9)	**2.11**	**1.09–4.09**	**0.028**	**1.98**	**1.00–3.95**	**0.052**
Dimensions^i^								
Reality distortion	4.0 (2.9)	3.3 (2.6)	**0.91**	**0.83–0.99**	**0.031**	**0.91**	**0.83–1.00**	**0.052**
Negative	1.3 (1.9)	1.2 (1.9)	0.97	0.86–1.10	0.645	0.97	0.85–1.10	0.646
Disorganised	0.6 (0.7)	0.7 (0.9)	1.20	0.91–1.58	0.207	1.24	0.92–1.65	0.152
Mania	1.2 (2.2)	2.0 (3.1)	**1.12**	**1.02–1.23**	**0.014**	**1.14**	**1.03–1.25**	**0.010**
Depression	1.2 (1.8)	1.4 (1.9)	1.07	0.94–1.22	0.286	1.02	0.89–1.17	0.802
Symptom severity^i^	8.3 (4.8)	8.7 (5.0)	1.02	0.97–1.07	0.546	1.01	0.96–1.07	0.607

a Adjusted for, as appropriate, centre, sex, age (as continuous variable), and ethnicity

b Missing, 58.

c Missing, 75.

d Missing, 8.

e Missing, 21.

f Missing, 75.

g Note, no variable for percentage of time on medication during first year following contact was associated with recovery.

h Missing, 36.

i Missing, 7.

Functional recovery at 10 years (GAF ⩾ 60) was achieved by 117 (40.9% of 286). Only a minority had been in paid work for over 75% of the follow-up period (34 of 290; 11.7%), and a minority for between 25% and 75% of the follow-up (48; 16.6%), with the overwhelming majority employed for <25% (208, 71.7%). Similarly, at follow up only 22.8% (66) had been in paid work. Most individuals were single throughout follow-up (218, 71.9%) and at follow-up (205, 67.7%).

### Predictors of symptom and functional recovery at 10 years

As noted, we examined the effects of four blocks of factors on symptom (Table 2) and functional (Table 4) recovery at 10-years: demographic; social; clinical; and early symptom course (remission at 12-weeks). Effects are shown before and after adjustment for age, gender, study centre, and ethnicity. We additionally performed these analyses only including the subset of patients with a diagnosis of schizophrenia (See Supplementary Table).

### Predictors of symptom recovery at 10 years

Early symptom course (remission at 12-weeks) strongly predicted recovery (adj OR 4.47; CI 2.60–7.67). Other variables associated with recovery (at *p* < 0.05) were a diagnosis of mania with psychosis (adj OR 3.22; CI 1.54–6.73) or of depression with psychosis (adj OR 1.98; CI 1.00–3.95); and on the dimension scales, a high symptom score on mania (adj OR 1.14; CI 1.03–1.25) or a low symptom score on reality distortion (adj OR 0.91; CI 0.83–1.00). Recovery was less likely (*p* ⩽ 0.05) among those with high scores for social disadvantage (score = 3, adj OR 0.40; CI 0.18–0.87; score = 4, adj OR 0.29; CI 0.13–0.67) and non-white British ethnicity (adj OR 0.43; CI 0.25–0.76) (Table 2).

When we further adjusted for all other variables retained in our models (as detailed above), strong associations with symptom recovery at 10 years (*n* = 213) remained for early symptom course (remission at 12-weeks) (adj OR 3.33; CI 1.72–6.43), a diagnosis of psychotic depression (adj OR 2.68; CI 1.02–7.05), and non-white British ethnicity (adj OR 0.44; CI 0.21–0.91) (Table 3). Our final model explained around 15% of the variance in symptom recovery and correctly predicted 61.0% with symptom recovery.

**Table 3.** Symptomatic Recovery at 10 years, adjusted model (number included in analyses = 213)

	Adj. OR^a^	95% CI	*p*	Adj. OR^b^^,^^c^^,^^d^	95% CI	*p*
Demographic						
Ethnicity						
White British	1.00	–	–	1.00	–	–
Other	**0.43**	**0.22–0.85**	**0.015**	**0.44**	**0.21–0.91**	**0.027**
Social						
Index of social disadvantage						
0, 1	1.00	–	–	1.00	–	–
2	0.63	0.26–1.49	0.289	0.79	0.31–2.03	0.624
3	0.54	0.24–1.23	0.144	0.70	0.28–1.74	0.444
4	**0.29**	**0.12–0.69**	**0.006**	0.46	0.18–1.18	0.107
Early clinical course						
Remission at 12 weeks						
No	1.00	–	–	1.00	–	–
Yes	**3.92**	**2.13–7.21**	**<0.001**	**3.33**	**1.72–6.43**	**<0.001**
Clinical						
Diagnosis						
Non-affective	1.00	–	–	1.00	–	–
Mania	**4.09**	**1.71–9.78**	**0.002**	1.74	0.48–6.27	0.395
Depression	**2.84**	**1.17–6.89**	**0.021**	**2.68**	**1.02–7.05**	**0.045**
Dimensions						
Reality distortion	**0.89**	**0.80–0.98**	**0.022**	0.94	0.84–1.05	0.289
Mania	**1.11**	**1.00–1.23**	**0.047**	1.03	0.89–1.20	0.704

a Adjusted for centre, sex, age (as continuous variable), and ethnicity

b Adjusted for centre, sex, age (as continuous variable), and ethnicity, and all other variables in table

c Pseudo *r*^2^ 0.15 (i.e. approximately 15% of variance explained by variables in model)

d Percentage with symptom recovery correctly predicted, 61.0%

### Predictors of functional recovery at 10 years

Early symptom course (remission at 12-weeks) strongly predicted functional recovery (i.e. GAF score >60) (adj OR 4.43; CI 2.46–7.98) (Table 4). The following also strongly predicted functional recovery (at *p* ⩽ 0.05): study centre (i.e. Nottingham *v.* south-east London) (OR 1.88; CI 1.02–3.48); being female (adj OR 2.19; CI 1.30–3.68); having a diagnosis of mania with psychosis (adj OR 11.28; CI 4.40–28.92) or depression with psychosis (adj OR 2.64; CI 1.20–5.85); high mania symptoms scores (adj OR 1.26; CI 1.12–1.41); and low negative symptom scores (adj OR 0.78; CI 0.66–0.92).

**Table 4.** Functional Recovery (i.e. GAF-D ⩾ 60) at 10 years (unless specified, number included in analyses = 286)

	Not recovered (*N* = 169) *N* (%) or mean (s.d.)	Recovered (*N* = 117) *N* (%) or mean (s.d.)	Unadj.OR	95% CI	*p*	Adj.OR (1)^a^	95% CI	*p*
Demographic								
Study Centre								
London	122 (72.2)	61 (52.1)	1.00	–	–	1.00	–	–
Nottingham	47 (27.8)	56 (47.9)	**2.38**	**1.45–3.91**	**0.001**	**1.88**	**1.02–3.48**	**0.043**
Sex								
Men	105 (62.1)	53 (45.3)	1.00	–	–	1.00	–	–
Women	64 (37.9)	64 (54.7)	**1.98**	**1.23–3.20**	**0.005**	**2.19**	**1.30–3.68**	**0.003**
Age								
16–29	93 (55.0)	58 (49.6)	1.00	–	–	1.00	–	–
30–64	76 (45.0)	59 (50.4)	1.24	0.78–2.00	0.364	1.29	0.77–2.16	0.325
Ethnicity								
White British	50 (29.6)	61 (52.1)	1.00	–	–	1.00	–	–
Other	119 (70.4)	56 (47.9)	**0.39**	**0.24–0.63**	**<0.001**	**0.50**	**0.28–0.89**	**0.018**
Social								
Index of social disadvantage^b^								
0, 1	16 (11.9)	38 (38.0)	1.00	–	–	1.00	–	–
2	34 (25.4)	25 (25.0)	**0.31**	**0.14–0.68**	**0.003**	**0.35**	**0.15–0.82**	**0.015**
3	43 (32.1)	21 (21.0)	**0.21**	**0.09–0.45**	**<0.001**	**0.22**	**0.09–0.52**	**0.001**
4	41 (30.6)	16 (16.0)	**0.16**	**0.07–0.37**	**<0.001**	**0.18**	**0.07–0.44**	**<0.001**
Loss, separation from parent^c^								
None	58 (45.7)	53 (55.8)	1.00	–	–	1.00	–	–
Loss (parent died)	12 (9.5)	8 (8.4)	0.73	0.28–1.92	0.524	0.52	0.18–1.54	0.239
Separation from parent	57 (44.9)	34 (35.8)	0.65	0.37–1.15	0.139	0.73	0.39–1.38	0.336
Education^d^								
University	12 (7.5)	20 (17.2)	1.00	–	–	1.00	–	–
Further	49 (30.4)	30 (25.9)	**0.37**	**0.16–0.86**	**0.021**	0.53	0.22–1.31	0.171
GCSE	39 (24.2)	35 (30.2)	0.54	0.23–1.26	0.153	0.85	0.34–2.13	0.726
No qualifications	61 (37.9)	31 (26.7)	**0.30**	**0.13–0.70**	**0.005**	**0.40**	**0.16–0.99**	**0.047**
Early symptom course								
Remission at 12 weeks^e^								
No	118 (79.2)	48 (44.0)	1.00	–	–	1.00	–	–
Yes	31 (20.8)	61 (56.0)	**4.84**	**2.80–8.36**	**<0.001**	**4.43**	**2.46–7.98**	**<0.001**
% time on med, first 3 months^f^^,^^g^	65.5 (39.4)	70.1 (37.1)	1.00	1.00–1.01	0.363	1.00	0.99–1.01	0.884
Clinical								
Mode of onset^h^								
Acute	51 (35.2)	58 (54.7)	1.00	–	–	1.00	–	–
Insidious	94 (64.8)	48 (45.3)	**0.45**	**0.27–0.75**	**0.002**	**0.41**	**0.23–0.73**	**0.021**
DUP^h^								
Short	68 (42.0)	71 (61.2)	1.00	–	–	1.00	–	–
Long	94 (58.0)	45 (38.8)	**0.46**	**0.28–0.75**	**0.002**	**0.41**	**0.24–0.71**	**0.001**
Diagnosis								
Non-affective	145 (85.8)	63 (53.9)	1.00	–	–	1.00	–	–
Mania	7 (4.1)	33 (28.2)	**10.85**	**4.56–25.84**	**<0.001**	**11.28**	**4.40–28.92**	**<0.001**
Depression	17 (10.1)	21 (17.9)	**2.84**	**1.41–5.75**	**0.004**	**2.64**	**1.20–5.85**	**0.016**
Dimensions^i^								
Reality distortion	3.9 (3.0)	3.5 (2.5)	0.95	0.87–1.03	0.225	0.99	0.90–1.09	0.864
Negative	1.5 (2.2)	0.9 (1.4)	**0.83**	**0.72–0.96**	**0.011**	**0.78**	**0.66–0.92**	**0.003**
Disorganised	0.7 (0.9)	0.7 (0.9)	1.06	0.81–1.38	0.686	1.04	0.78–1.40	0.775
Mania	1.0 (2.0)	2.5 (3.3)	**1.25**	**1.12–1.39**	**<0.001**	**1.26**	**1.12–1.41**	**<0.001**
Depression	1.2 (1.8)	1.3 (1.8)	1.04	0.91–1.20	0.532	1.02	0.88–1.19	0.757
Symptom severity^i^	8.3 (4.5)	8.9 (4.9)	1.03	0.98–1.09	0.262	1.04	0.98–1.10	0.188

a Adjusted for, as appropriate, centre, sex, age (as continuous variable), and ethnicity.

b Missing, 52.

c Missing, 64.

d Missing, 9.

e Missing, 28.

f Missing, 58.

g Note, no variable for percentage of time on medication during first year following contact was associated with recovery.

h Missing, 35.

i Missing, 22.

Functional recovery was less likely among those of non-white British ethnicity (adj OR 0.50; CI 0.28–0.89); those with no educational qualifications (adj OR 0.40; CI 0.16–0.99); those with insidious mode of onset (adj OR 0.41; CI 0.23–0.73); and those with longer DUP (adj OR 0.41; CI 0.24–0.71) (Table 4). There was a strong association between recovery and social disadvantage, such that with each additional disadvantage, there was a progressively lower likelihood of recovery (score = 2, OR 0.35; CI 0.15–0.82; score = 3, OR 0.22; CI 0.09–0.52; and score = 4, OR 0.18; CI 0.07–0.44).

When we further adjusted for all other variables retained in our models (as detailed above), strong associations with functional recovery at 10 years (*n* = 213) remained for early symptom course (remission at 12-weeks) (adj OR 2.75; CI 1.23–6.11), study centre (i.e. Nottingham *v.* south-east London) (adj OR 3.23; CI 1.25–8.30), and a diagnosis of mania with psychosis (adj OR 8.17; CI 1.61–41.42) (Table 5). Our final model explained around 32% of the variance in functional recovery and correctly predicted 73.6% with functional recovery.

**Table 5.** Functional Recovery at 10 years, adjusted model (number included in analyses = 197)

	Adj. OR^a^	95% CI	*p*	Adj. OR^b^^,^^c^^,^^d^	95% CI	p
Demographic						
Centre						
London	1.00	–	–	1.00	–	–
Nottingham	**3.09**	**1.42–6.71**	**0.004**	**3.23**	**1.25–8.30**	**0.015**
Sex						
Men	1.00	–	–	1.00	–	–
Women	**2.65**	**1.42–4.94**	**0.002**	1.81	0.85–3.88	0.126
Ethnicity						
White British	1.00	–	–	1.00	–	–
Other	**0.56**	**0.28–1.14**	**0.110**	0.52	0.22–1.25	0.143
Education						
University	1.00	–	–	1.00	–	–
Further	**0.29**	**0.09–0.95**	**0.040**	0.36	0.87–1.49	0.158
GCSE	0.53	0.16–1.72	0.292	1.44	0.35–5.88	0.613
No qualifications	**0.20**	**0.06–0.64**	**0.007**	0.47	0.12–1.90	0.291
Social						
Index of social disadvantage						
0, 1	1.00	–	–	1.00	–	–
2	**0.37**	**0.15–0.94**	**0.036**	0.53	0.18–1.56	0.248
3	**0.28**	**0.11–0.70**	**0.007**	0.38	0.12–1.15	0.086
4	**0.19**	**0.07–0.48**	**0.001**	0.38	0.12–1.21	0.101
Early symptom course						
Remission at 12 weeks						
No	1.00	–	–	1.00	–	–
Yes	**4.04**	**2.10–8.00**	**<0.001**	**2.75**	**1.23–6.11**	**0.013**
Clinical						
DUP						
Short	1.00	–	–	1.00	–	–
Long	**0.35**	**0.18–0.66**	**0.001**	0.52	0.24–1.13	0.098
Diagnosis						
Non-affective	1.00	–	–	1.00	–	–
Mania	**14.39**	**4.50–46.02**	**<0.001**	**8.17**	**1.61–41.42**	**0.011**
Depression	**2.76**	**1.04–7.31**	**0.042**	2.83	0.88–9.07	0.080
Dimensions						
Negative	0.87	0.73–1.05	0.161	0.86	0.69–1.09	0.213
Mania	**1.20**	**1.07–1.35**	**0.002**	1.00	0.84–1.19	0.988

*Note:* Mode of onset and DUP are strongly associated; therefore, only one (DUP) was included in final model.

a Adjusted for centre, sex, age (as continuous variable), and ethnicity.

b Adjusted for centre, sex, age (as continuous variable), and ethnicity, and all other variables in table.

c Pseudo *r*^2^ 0.32 (i.e., approximately 32% of variance explained by variables in model).

d Percentage with functional recovery correctly predicted, 73.6%.

## Discussion

This is the first long-term, epidemiological, naturalistic follow-up that has investigated the specific role of early remission and other predictors of outcome across multiple domains in a cohort of individuals following their first episode of psychosis. Our main finding is that symptom remission at 12-weeks is a strong predictor of both symptom and functional recovery at 10-years. Furthermore, we found that diagnosis at first onset is a strong predictor of recovery: depressive psychosis was predictive of good symptom recovery and manic psychosis was highly predictive of good functional recovery. Finally, being from the Nottingham study centre was predictive of good functional recovery. Overall, our final models explained more of the variance in functional than symptom outcomes, although for both the percentage explained was relatively low. Clearly, other unmeasured factors also contribute to variations in recovery in psychosis.

This study has a number of strengths. It used an incidence cohort of first episode psychosis patients who presented to any psychiatric service. This reduced the chances of only including patients with a particularly illness type, as is often the case for in-patient populations or those drawn only from early intervention services. The study naturalistic approach makes this sample as unbiased as possible. Finally, the study is unique in having evaluated a broad range of predictors across multiple domains over time, allowing the description of the heterogeneous course trajectories of psychosis. A major strength is the reporting of recovery across a range of domains, which has yielded evidence that predictors of symptom and functional recovery differ and are somewhat independent of each other.

Some limitations should be acknowledged. As with all longitudinal evaluations, we cannot exclude the potential for selection and information bias arising from, respectively, loss to follow-up and missing or inaccurate data. However, we were able to evaluate the symptom and functional status of over 90% of the cohort. We found no strong evidence of systematic bias when we compared those with some information available with those without (Morgan *et al*., 2014), suggesting that attrition is unlikely to have affected findings. The potential for information bias should also be considered. We made use of all possible sources of information, and clinical ratings were made by consensus. To limit the potential variability in the extent and quality of information available, we only made ratings of the presence or absence of symptoms on the basis of clear and definite information. Also, we only considered lifetime substance use and did not separately consider patterns of use and abstinence following FEP. Finally, completeness of information was inevitably less for those not re-interviewed. However, we found that symptom course and outcome were better in the Nottingham sample, while, at the same time, cases in Nottingham were less likely to be re-interviewed.

This is the first time that a role for early symptom remission (defined with an operationalized set of criteria) has been established as important in the *long-term* outcome of any psychosis. This finding advances evidence from shorter longitudinal studies that early symptom remission is associated with better symptomatic outcome (Emsley *et al*., 2006; Verma *et al*., 2012). It adds to existing evidence that a shorter proportion of time spent experiencing symptoms in the first years of illness (Harrison *et al*., 2001) is associated with better long-term outcome, and importantly, it demonstrates that this applies to both symptom and functional domains.

This crucial role for early remission could be explained in multiple ways. These first 12 weeks may be the time required to receive at least one adequate trial of pharmacological treatment. While it is theoretically possible that those individuals who did not achieve early remission were those who had received no or ineffective treatment, our data suggest this was not the case. There were no differences in exposure to antipsychotics between individuals who did and did not remit at 12-weeks. It is also possible that these early responders represent a subgroup more likely to respond to treatment. This is consistent with emerging evidence that between 10 and 20% of first episode patients show rapid response to the first antipsychotic received (Agid *et al*., 2013). In our study, these individuals follow a more benign illness trajectory. They may warrant a less assertive management approach after the resolution of the first episode. Conversely, identifying non-responders early in treatment could enable clinicians to consider alternative treatment options in order to find the earliest possible effective treatment (Kinon *et al*., 2010).

Our data support early management in first episode psychosis, which aims not only to reduce DUP, but also to promptly reduce symptom severity, to minimize the time spent with psychotic symptoms. In our sample, DUP was not a predictor of poor symptom recovery, but rather a predictor of poor functional recovery at 10-years. Our interpretation of this finding is that once patients receive antipsychotic treatment, their ability to respond is the key predictor of future symptom recovery, independently of DUP. In turn, a longer DUP may affect their ability to function in social and occupational spheres, and where these deficits are engrained over time, they persist even despite symptomatic improvement. Therefore, while antipsychotic treatment is an important part of early first episode psychosis management, it remains only one aspect of the holistic care that can be provided, which would include also non-pharmacological interventions such as for example psychoeducation and family work.

Interestingly, one-third of patients who initially remitted did not recover in the long-term. This heterogeneity of illness course may reflect the interplay between external factors and the pathophysiology of the disorder, which over time generates multiple possible trajectories. For example, intervening life events, stress, substance use, ongoing symptoms, family support, vocational opportunities, compliance with pharmacological and other treatments may independently contribute to shaping illness course after the initial period (Racenstein *et al*., 2002; Petersen *et al*., 2005; Miller, 2008; Jordan *et al*., 2014; Colizzi *et al*., 2016; Weibell *et al*., 2017).

Another important predictor of recovery at 10-years was a diagnosis of depressive or manic psychosis. To our knowledge, this is the first time that differences in outcome have been investigated for affective psychosis diagnostic subtypes. Depressive psychosis predicted better symptom recovery, while mania strongly predicted better functional recovery. Affective disorders, in general, have been reported to be predictive of a better outcome in longitudinal studies of duration 15–25 years (Marneros *et al*., 1990; Bottlender *et al*., 2010; Henry *et al*., 2010). Our findings align with long-term follow-ups of major depressive disorder (psychotic and non-psychotic), suggesting that psychosocial disability is associated with severity of depressive/bipolar 1 symptoms (Judd *et al*., 2005). In our study, those patients with a manic presentation were symptomatic for a shorter period than those with schizophrenia, and so were more likely to achieve 12-week remission. However, manic psychosis was an additional highly independent predictor of functional recovery. Good functional recovery was also predicted by being from the Nottingham study centre. There was evidence that functional outcomes were better, on average, for patients in Nottingham. While we do not know the reasons for this, it is tantalising to speculate that the factors related to urbanicity that increase risk of psychosis onset also impact on its outcome. Finally, since patients with schizophrenia are those more likely to be considered for long term maintenance antipsychotic treatment, we repeated the analyses including only participants with this diagnosis. We find that even considering only this group, all findings are in the same direction as the main findings, though inevitably with some loss of power likely due to the smaller sample sizes (See online Supplementary Table S1). These additional analyses confirmed that our main finding that early remission is the best predictor of recovery stands.

While other demographic, clinical, and social factors were considered, their predictive power in the regression models of 10-year recovery was modest. The only notable predictor of symptom recovery, in addition to early remission and depressive psychosis diagnosis, was White British ethnicity. This is consistent with our previous findings in this sample, that black Caribbean individuals experience worse clinical, social, and service use outcomes, and black African individuals worse social and service use outcomes (Morgan *et al*., 2017). There is growing evidence that substance use is associated with poorer outcomes following FEP (Lambert *et al*., 2005; Petersen *et al*., 2005), but it did not independently predict outcome in our sample, possibly because of the method we used to evaluate use. Ideally, substance use throughout follow-up would be assessed by serial assessments verified by toxicology. However, this may also be due to the fact that we included individuals presenting with FEP throughout adulthood (16–65 years), in contrast to other studies that included younger individuals in whom substance use is more prevalent [e.g. (15–29 years) (Lambert *et al*., 2005); (16–40 years) (Petersen *et al*., 2005)].

In conclusion, remission at 12-weeks should be used to inform clinical management following first episode psychosis. It may represent a useful means of stratifying patients into treatment protocols reflecting likely subsequent course. While the diagnosis may also have a predictive role, with depressive and manic presentations being predictive of long-term clinical and functional recovery respectively, our data suggest that prediction of long-term outcome from the first episode of any psychosis remains extremely challenging.

## Supplementary material

For supplementary material accompanying this paper visit https://doi.org/10.1017/S0033291719001399. click here to view supplementary material
