# Supplementary material for: Symptom remission at 12-weeks strongly predicts long-term recovery from the first episode of psychosis
Source: Psychol Med. 2019 Jul 25;50(9):1452–62. doi: 10.1017/S0033291719001399 (PMC7385193; doi:10.1017/S0033291719001399)
Supplement: Supplementary file 1 [file S0033291719001399sup001.docx]

**Supplementary Table. Symptomatic Recovery at 10 years, adjusted model (schizophrenia only: number included in analyses = 152)**

|  |  | **Adj. OR (1)** | **95% CI** | **p** | **Adj. OR (2)*†** | **95% CI** | **p** |
| --- | --- | --- | --- | --- | --- | --- | --- |
| **Demographic** | |  |  |  |  |  |  |
| Ethnicity | |  |  |  |  |  |  |
|  | White British | 1·00 | - | - | 1·00 | - | - |
|  | Other | 0.65 | 0.31-1.40 | 0.273 | 0.69 | 0.30-1.58 | 0.379 |
| **Social** | |  |  |  |  |  |  |
| Index of social disadvantage | |  |  |  |  |  |  |
|  | 0, 1 | 1·00 | - | - | 1·00 | - | - |
|  | 2 | 0.94 | 0.33-2.68 | 0.913 | 0.90 | 0.30-2.75 | 0.858 |
|  | 3 | 0.85 | 0.31-2.32 | 0.750 | 0.81 | 0.28-2.39 | 0.716 |
|  | 4 | 0.44 | 0.16-1.23 | 0.117 | 0.46 | 0.16-1.37 | 0.162 |
| **Early clinical course** | |  |  |  |  |  |  |
| Remission at 12 weeks | |  |  |  |  |  |  |
|  | No | 1·00 | - | - | 1·00 | - | - |
|  | Yes | **4.12** | **2.00-8.64** | **<0.001** | **3.97** | **1.89-8.33** | **<0.001** |
| **Clinical** | |  |  |  |  |  |  |
| Diagnosis | |  |  |  |  |  |  |
|  | Non-affective | - | - | - | - | - | - |
|  | Mania | **-** | **-** | **-** | - | - | - |
|  | Depression | **-** | **-** | **-** | **-** | **-** | **-** |
| Dimensions | |  |  |  |  |  |  |
|  | Reality distortion | 0.95 | 0.84-1.06 | 0.352 | 0.95 | 0.84-1.08 | 0.436 |
|  | Mania | 1.00 | 0.84-1.19 | 0.987 | 1.04 | 0.86-1.26 | 0.654 |

(1) Adjusted for centre, sex, age (as continuous variable), and ethnicity

(2) Adjusted for centre, sex, age (as continuous variable), and ethnicity, and all other variables in table

* pseudo r^2^ 0·10 (i.e., approximately 10% of variance explained by variables in model)

**Supplementary Table. Functional Recovery at 10 years, adjusted model (schizophrenia only: number included in analyses = 142)**

|  |  | **Adj. OR (1)** | **95% CI** | **p** | **Adj. OR (2)*†** | **95% CI** | **p** |
| --- | --- | --- | --- | --- | --- | --- | --- |
| **Demographic** | |  |  |  |  |  |  |
| Centre | |  |  |  |  |  |  |
|  | London | 1·00 | - | - | 1·00 | - | - |
|  | Nottingham | **2.53** | **1.05-6.10** | **0.039** | **2.78** | **0.94-8·24** | **0·065** |
| Sex | |  |  |  |  |  |  |
|  | Men | 1·00 | - | - | 1·00 | - | - |
|  | Women | 1.75 | 0.81-3.76 | 0·154 | 1·53 | 0·64-3.66 | 0·344 |
| Ethnicity | |  |  |  |  |  |  |
|  | White British | 1·00 | - | - | 1·00 | - | - |
|  | Other | **0·44** | **0·19-1·00** | **0·049** | 0·47 | 0·18-1·22 | 0·120 |
| Education | |  |  |  |  |  |  |
|  | University | 1·00 | - | - | 1·00 | - | - |
|  | Further | 0.40 | 0.07-2.14 | 0.282 | 0.42 | 0.07-2.52 | 0.344 |
|  | GCSE | 1.18 | 0.23-5.95 | 0.840 | 1.61 | 0.29-8.95 | 0.583 |
|  | No qualifications | 0.54 | 0.11-2.76 | 0.459 | 0.73 | 0.12-4.31 | 0.728 |
| **Social** | |  |  |  |  |  |  |
| Index of social disadvantage | |  |  |  |  |  |  |
|  | 0, 1 | 1·00 | - | - | 1·00 | - | - |
|  | 2 | 0·73 | 0·24-2.28 | 0·590 | 0.79 | 0.23-2.75 | 0.713 |
|  | 3 | 0·51 | 0·16-1.64 | 0·261 | 0.53 | 0.15-1.95 | 0.342 |
|  | 4 | 0·35 | 0·11-1.12 | 0·077 | 0.41 | 0.11-1.47 | 0.170 |
| **Early symptom course** | |  |  |  |  |  |  |
| Remission at 12 weeks | |  |  |  |  |  |  |
|  | No | 1·00 | - | - | 1·00 | - | - |
|  | Yes | **2.70** | **1.22-5.97** | **0·014** | **2·56** | **1·07-6·13** | **0·035** |
| **Clinical** | |  |  |  |  |  |  |
| DUP | |  |  |  |  |  |  |
|  | Short | 1·00 | - | - | 1·00 | - | - |
|  | Long | 0·47 | 0·22-1.04 | 0·063 | 0·57 | 0·24-1.36 | 0·209 |
| Diagnosis | |  |  |  |  |  |  |
|  | Non-affective | - | - | - | - | - | - |
|  | Mania | **-** | **-** | **-** | **-** | **-** | **-** |
|  | Depression | **-** | **-** | **-** | - | - | - |
| Dimensions | |  |  |  |  |  |  |
|  | Negative | 0·85 | 0·68-1·08 | 0·192 | 0·85 | 0·65-1·10 | 0·218 |
|  | Mania | 1·07 | 0.89-1·29 | 0·463 | 1·08 | 0·88-1·33 | 0·440 |

Note, Mode of onset and DUP are strongly associated; therefore, only one (DUP) was included in final model

(1) Adjusted for centre, sex, age (as continuous variable), and ethnicity

(2) Adjusted for centre, sex, age (as continuous variable), and ethnicity, and all other variables in table

* pseudo r^2^ 0·19 (i·e·, approximately 19% of variance explained by variables in model)
